# Deep-Learning-Based Real-Time Road Traffic Prediction Using Long-Term Evolution Access Data

**DOI:** 10.3390/s19235327

**Published:** 2019-12-03

**Authors:** Byoungsuk Ji, Ellen J. Hong

**Affiliations:** 1 Convergence Laboratory, KT R&D Center, Seoul 06763, Korea; bs88.ji@kt.com; 2 Department of Computer and Telecommunication Engineering, Yonsei University, Wonju-si 26493, Korea

**Keywords:** road traffic prediction, LTE access data, cellular phones, long short-term memory (LSTM), deep learning

## Abstract

In this paper, we propose a method for deep-learning-based real-time road traffic predictions using long-term evolution (LTE) access data. The proposed system generates a road traffic speed learning model based on road speed data and historical LTE data collected from a plurality of base stations located within a predetermined radius from the road. Real-time LTE data were the input for the generated learning model in order to predict the real-time speed of traffic. Since the system was developed using a time-series-based road traffic speed learning model based on LTE data from the past, it is possible for it to be used for a road where the environment has changed. Moreover, even on roads where the collection of traffic data is invalid, such as a radio shadow area, it is possible to directly enter real-time wireless communications data into the traffic speed learning model to predict the traffic speed on the road in real time, and in turn, raise the accuracy of real-time road traffic predictions.

## 1. Introduction

Recently, there has been a rapid diversification of traffic information services, and vehicle navigation is one of the most representative services. A navigation system receives information on the current location of a vehicle and provides information on the route and arrival time from a global positioning system (GPS). Since the user requires a high reliability for the arrival time, it is critical to increase the reliability of traffic information. In order to do so, it is important to include as much traffic information as possible for the prediction of traffic conditions ahead. However, because there are roads where probe vehicles do not pass by and where speed detectors are not installed, shadow roads are somewhat inevitable. Typically, historical statistics-based pattern data are used for these shadow roads. Still, such historical statistics-based pattern data cannot reflect the current traffic conditions, in turn creating the possibility that road traffic speed information will be completely different from actual road traffic speeds. Therefore, this paper proposes a method to obtain more accurate predictions of traffic speeds on the road by using long-term evolution (LTE) access data from each base station instead of individual driver’s access information, which avoids issues of privacy infringement. To raise the performance of a road traffic speed learning model, this paper suggests a way to extract only data exclusively generated by drivers from the access data of each base station by considering the nature of wireless connections.

## 2. Related Works

The most widely used parameters for creating traffic information using mobile communications data have included cellular phone locations, cell dwelling times, signal strength, and handover data. Cellular phone position-based traffic information generation uses the GPS from among the various sensors of a cellular phone to predict speed, congestion, and other conditions on the road [1,2,3,4,5,6,7]. There is also a method of predicting the volume of traffic based on the hours of cellular phone use [8,9,10,11]. However, these methods have the weakness of increasing battery consumption due to the frequent sampling of the GPS in the driver’s cellular phone. Chandrasekaran et al. [12,13] estimated the average speed of traffic by using the signal strength of cellular phones, comparing the signal strength trace on the cellular phones with the known trace of roads and calculating the average traffic speed. However, even though this method could cover most arterial roads, it fails to trace the changes in traffic speed in an accurate manner. Another method is a handover type of speed estimation. It searches for a base station that consequently hands over a large number of users and predicts the traffic speed on the road in real time by calculating the difference in access times between two consecutive base stations [14,15,16,17,18,19,20]. However, these methods have an issue owing to large system loads from the search for base stations, as well as from calculating the differences in distance and access times between them. Moreover, unless the hand over is always made at a specific point, it could be highly reliant on just a few users, which could be outliers or stationary drivers, and the predicted road traffic speeds could greatly vary. In order to properly address such weaknesses in the aforementioned methods, this paper suggests using wireless communications access logs, collected in real time, and only data taken exclusively from drivers, considering the nature of wireless connections.

## 3. Correlation Analysis of LTE Access Data and Traffic Information

**Figure 1:**
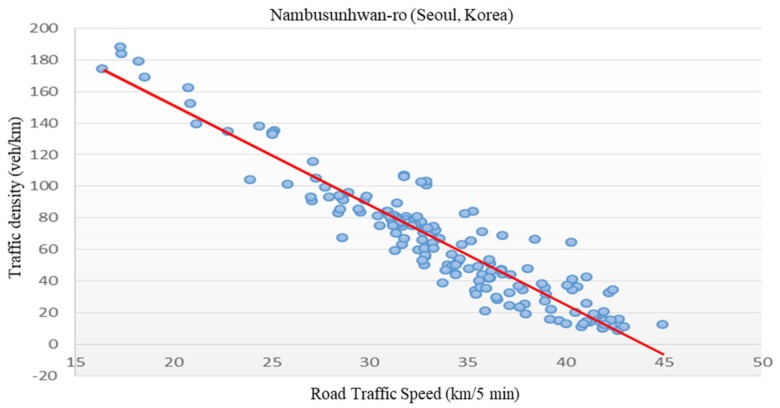
Speed–density correlation analysis of Nambusunhwan-ro in Seoul (Korea).

The purpose of this paper was to analyze correlations with traffic density to identify whether it is meaningful to generate traffic information using an amount of LTE access data. However, collecting traffic volume or density directly is a hurdle that has always been identified in the field of transportation. Fortunately, Seoul collects traffic density information on several major roads and makes this data public. In this paper, as a result of analyzing the relationship between traffic density information and speed information acquired from public data, linear characteristics were confirmed, as shown in Figure 1. This fits with Greenshield’s model [21] among the theoretical models providing correlations between traffic volume–speed–density, as defined in traffic engineering.

**Figure 2:**
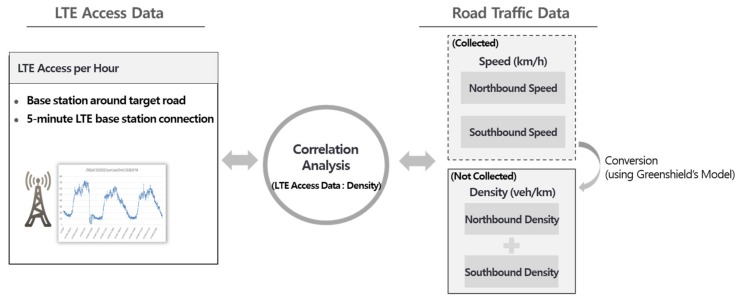
Conversion of speed into road density for the correlation analysis between the long-term evolution (LTE) access and road traffic.

To analyze the relationship between the LTE access volume and traffic information, traffic speed information was converted into traffic density. By reflecting the nature of the actual road environment and traffic flow, the volume of traffic flow for the northbound lane was combined with that of the southbound lane for the analysis, as shown in Figure 2. This paper analyzed the correlation between the converted density data and the LTE access data amount by applying the speed data collected using the Greenshield’s model [21].

**Figure 3:**
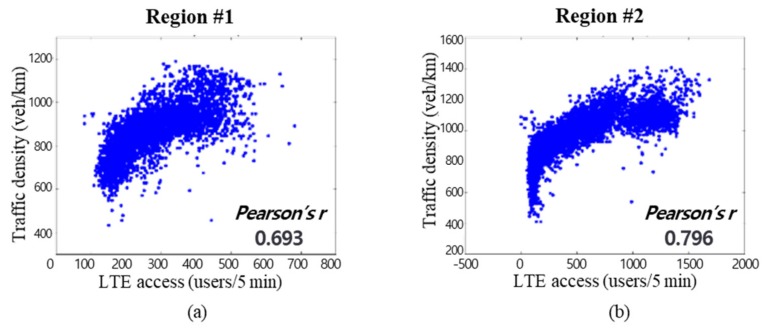
Correlation analysis of the traffic density and LTE access.

Figure 3 shows the findings from the analysis of the relationship between the LTE traffic volume and the actual speed–density data, in units of time, obtained by Topis [22], a Seoul traffic information system, on the Seoul Nambusunhwan-ro for one week (1–7 August 2017). As shown in Figure 3, generating traffic information using an amount of LTE access data was meaningful.

## 4. Deep-Learning-Based Real-Time Road Traffic Prediction System Using LTE Access Data Preprocessing

### 4.1. Configuration of the System

**Figure 4:**
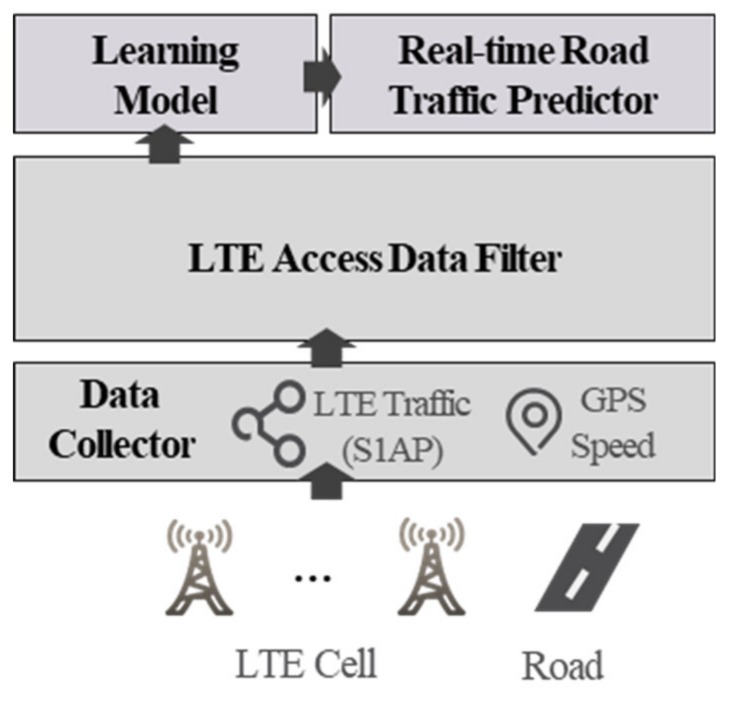
LTE traffic-based road traffic prediction system.

Figure 4 presents the configuration of a real-time road traffic prediction using LTE access data. A data collector collected wireless communication access data and GPS-based 5-minute road speeds from multiple base stations. Wireless access data was used as the input to the learning model, and GPS speed was used as the ground truth value for the output of the learning model. The wireless communication access data used in this paper was not data that could be collected or accessed by the public. The authors, as workers of a telecom provider, received and analyzed the data for the purpose of this research in a secure environment that was not connected to an external network. This telecom provider manages data in the form of a platform without personal information. This paper uses encrypted data, such as Secure Hash Algorithm (SHA)-256, for information that may have privacy or security issues, such as International Mobile Subscriber Identity (IMSI), rather than raw data. A data filter filtered the driver information from the collected data to raise the prediction accuracy of this learning model. Based on the filtered data and road traffic speed data, a traffic speed learning model was generated, and by entering the real-time wireless communications access data into the learning model, the real-time speed of the traffic was predicted. Each component of the system is described in detail in the following subsections.

### 4.2. Data Collector

**Figure 5:**
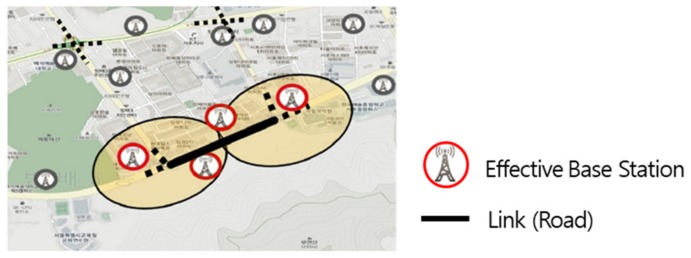
Effective base station selection.

Data are collected from select effective base stations to which drivers connect via wireless communications while driving by, and from LTE access data from these base stations in real time. As shown in Figure 5, a valid base station must be located within a predetermined radius from the road, and its antenna must be installed outside and directed toward the target road. Considering the nature of a base station where wireless communications access is distributed, two or more base stations must be selected. The collected access data included information on users who made attempts at wireless connection to each valid base station, as well as information about each effective base station.

**Figure 6:**
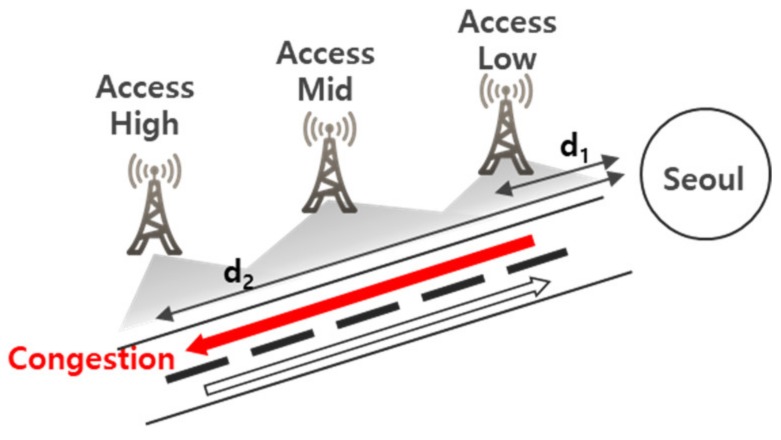
Classification of northbound and southbound directions according to the distribution of access.

By distinguishing the up and down directions of the roads based on the distribution of wireless communications traffic volume among multiple base stations and their locations, it is possible to predict the real-time traffic speed for each direction of the road. As shown in Figure 6, by using log data on the S1 application protocol (S1AP) collected from a plurality of base stations located within a predetermined radius of the road, it was possible to identify the distribution of wireless communications traffic volume among these base stations and to assign a direction on the road by examining the relationship between the location and distributed wireless communications traffic volume at each base station. For instance, among the effective base stations, the volume of wireless communications traffic could be higher at a base station with a distance d2 > d1 from Seoul, compared to a base station at distance d1 from Seoul. In such a case, the traffic flow of the southbound lane could be considered stagnant while the traffic flow of the northbound lane is smoother.

**Figure 7:**
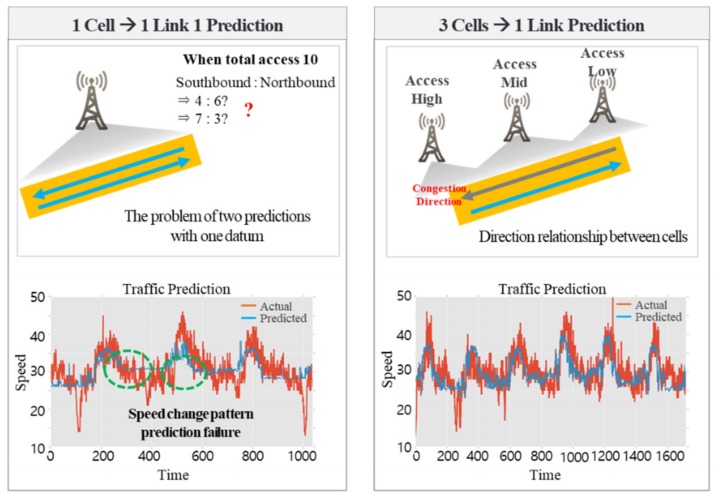
The results of assigning directionality from the relationship between the number of effective base stations and cells.

Figure 7 shows the results of the assignment of a direction based on the relationship between the numbers of effective base stations and cells. When predicting the traffic speed on a road line with one cell, it is difficult to predict the pattern of speed change since the speed of the northbound lane and of the southbound lane have to be predicted with a single datum. However, it is possible to predict a low-speed pattern by having three effective base stations and differentiating the road direction by using the distribution of wireless communications traffic volume. This cannot be done with a single base station.

### 4.3. LTE Data Preprocessing

As for the information accessed from a base station, it included both drivers driving by on a nearby road and non-drivers who were nearby. To raise the accuracy of the prediction of the road traffic speed, it was critical to filter out information on non-drivers, increasing the ratio of data on drivers, and to eliminate outliers. Generally, it is acceptable to consider the person whose dwelling time at one base station within one day is up to 15 minutes as a driver, assuming the driver generally drives along a highly congested one-kilometer section at a speed of, say, 4km/h, and to filter out others. However, this method has a critical drawback in that the dwelling time cannot be calculated in real time. In order to overcome this, we propose two methods. In addition, we suggest distinguishing between the volumes for incoming and outgoing traffic in order to raise the performance of the suggested deep learning model.

#### 4.3.1. LTE Access Frequency-Based Filtering

**Figure 8:**
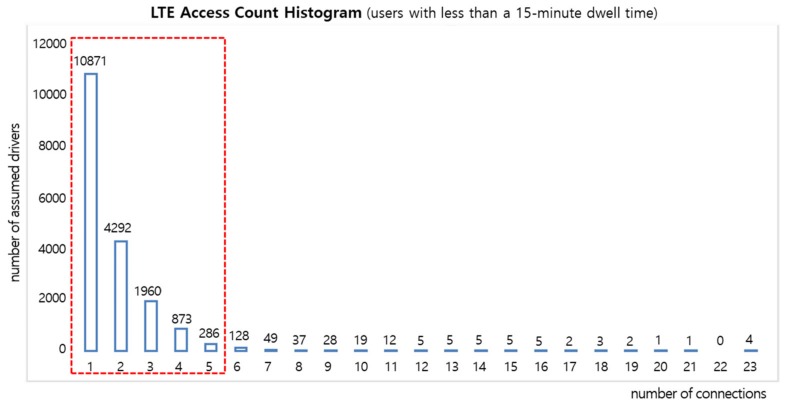
Correlation between the assumed driver and the number of LTE connections.

In the case of access data from pedestrians or residents who spend a large amount of time at a place adjacent to the road, there is a high possibility that it is not generated from a vehicle on the road; therefore, by excluding such data, it is possible to raise the accuracy of the road traffic speed predictions. Figure 8 shows the results of an analysis of the frequency of access to a certain base station by assumed drivers having a dwelling time of 15 minutes or less at one base station. It shows that 99% of 18,953 assumed drivers in one day accessed that base station up to five times. In other words, a person establishing LTE access to one base station more than five times is highly likely to be a non-driver. Thus, to distinguish between drivers and non-drivers, we suggest filtering the data for users who establish LTE access to one base station no more than five times.

**Figure 9:**
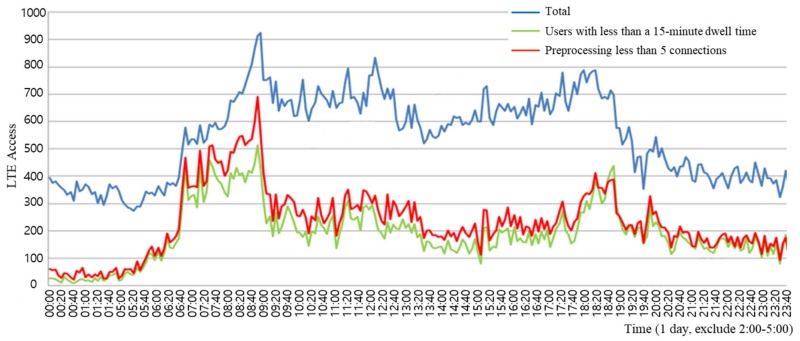
Comparison of assumed drivers and LTE access pattern with fewer than five accesses.

**Figure 10:**
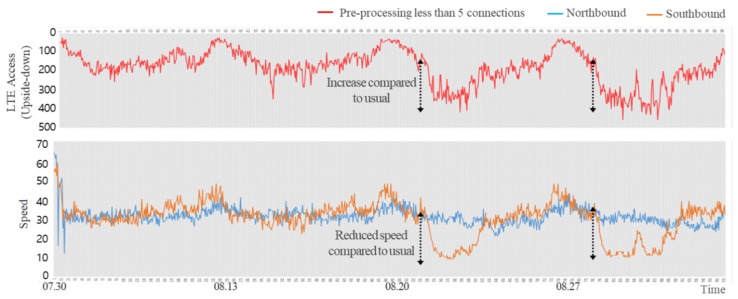
Comparison of preprocessed LTE connection data and road speed.

In addition, to eliminate data from residents, it is necessary to eliminate access data arising out of paging communication in the early morning. Paging communication refers to broadcast communications to regularly page a device in order to inspect the reception status and location of the device. Since access data arising out of paging communication is not access data generated from vehicles on the road, they should be eliminated to raise the accuracy of the road traffic speed predictions. Figure 9 shows that LTE access patterns of assumed drivers were very similar to users whose number of accesses were only up to five times. As shown in Figure 10, the comparison between the LTE access data, preprocessed with the suggested method, and the speed and pattern of the road shows the method of identifying assumed drivers based on the number of accesses.

#### 4.3.2. Filtering by Radio Resource Control (RRC) Link Classification of S1AP

S1AP handles the functions of paging and User Equipment (UE) context release, such as the UE context at the location of an evolved packet system (EPS) bearer, calls that are outgoing/ending, data service attempts, mobility, text messages, and push messages, among others. Table 1 shows the data schema of S1AP. We suggest filtering by the analysis of traffic volume based on the RRC classification, since it gets allocated with the resources from a network for a device using a communications service, as shown in Table 2.

**Figure 11:**
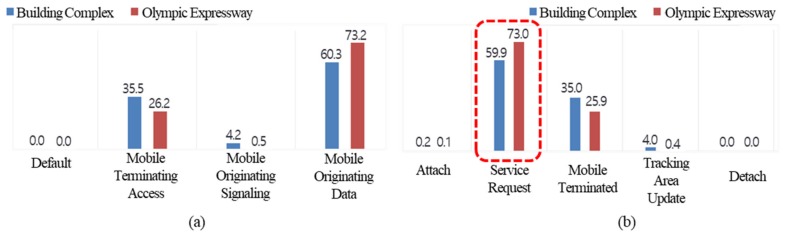
Analysis of driver-oriented characteristics: building complex versus the Olympic Expressway (in the driving environment, the number of wake-ups by an outgoing packet is more than 14%). (**a**) The number of connections according to the RRC connection type and (**b**) the number of connections according to the detail function type.

**Figure 12:**
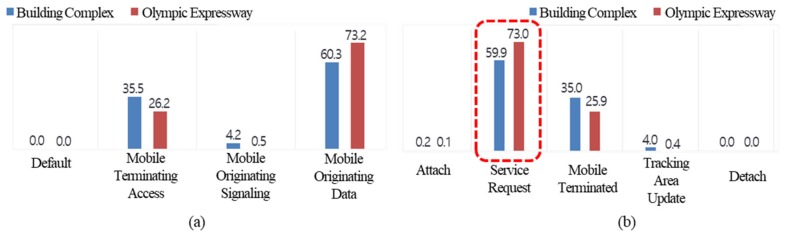
Analysis of abnormal situation characteristics: normal situations versus abnormal situations (in cases of congestion, the reception ratio of incoming data increased by 3%). (**a**) The number of connections according to the RRC connection type and (**b**) the number of connections according to the detail function type.

Figure 11 shows that terminals are often awakened by outbound packets since navigation or music services are mainly used while driving. As shown in Figure 12, the ratio of incoming data reception increases during congestion. Since the number of incoming and outgoing signals differs, depending on the road environment (such as congestion), it was necessary to differentiate them for the learning process.

#### 4.3.3. Filtering by QoS Class Index (QCI) Packets Under S1AP

**Figure 13:**
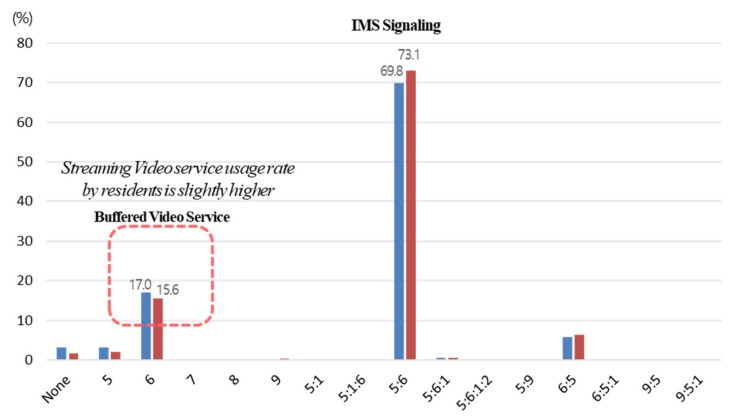
Analysis of driver-oriented characteristics according to the QCI value.

It was possible to eliminate data arising out of the access to services that could interfere with driving, such as a video streaming service, based on the index value for the service purpose of the communication protocol from a plurality of access data. The index value of the protocol, herein, was the QCI under S1AP, and it represents the priority of service importance in integers to assure the traffic quality. Table 3 shows a definition of the QCI. Figure 13 shows the results of filtering by using service packets that interfere with driving, such as a video streaming service. To reduce errors in learning and to increase the accuracy of prediction, it is necessary to eliminate data arising out of streaming services (QCI 6) that are not generally used when driving and that are highly used by residents, and to have data learning done mainly by QCI 5:6, which had a high explanation power during congestion. Passengers can watch videos or drivers can listen to music through YouTube streaming. However, in Korea, drivers are prohibited from watching videos. Even if illegal, many people will use streaming services while driving. It does not only matter where the driver’s LTE connection is collected, such as on the highway, but on residential roads, the number of streaming services by residents will be much higher than that of the drivers. In this paper, excluding streaming service access is done to strictly filter only drivers.

### 4.4. Road Traffic Speed Prediction Deep Learning Model

**Figure 14:**
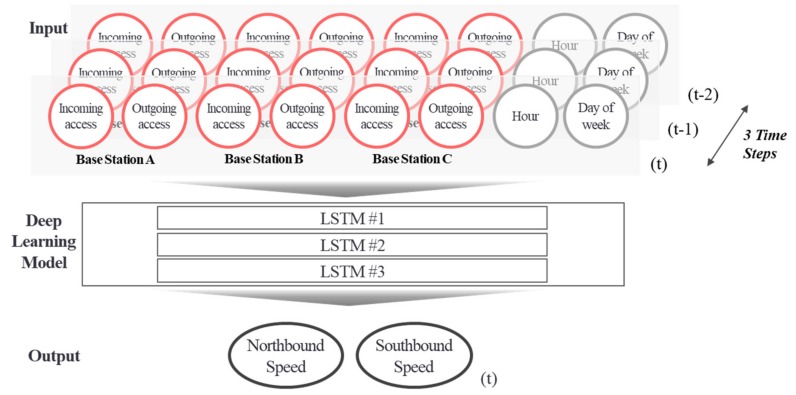
Deep learning model structure for traffic speed predictions.

Based on wireless communication access data and road speed data collected from multiple base stations, a road traffic speed learning model was generated. In other words, a time-series–based long short-term memory (LSTM) deep learning model was generated by using the wireless communications (S1AP) access volume from the past (t, t−1, t−2), which has gone through the suggested preprocessing, and the road speed data of the present (t), as shown in Figure 14. After entering the wireless communications access data (consisting of incoming and outgoing accesses for each effective base station) and communication influence weights, the time, day of the week, and traffic speeds were entered into the road traffic speed prediction deep learning model, we taught the model to output the current traffic speed for the up and down roads in the present. Communication influence weights varied depending on the scope of influence of each base station when there were multiple base stations on the road. By entering the collected real-time access data (real-time incoming and outgoing access volume) and the time and the day into the learning model, it was possible to estimate the speed of the northbound and southbound lanes. Twenty percent of the data set was put aside to become the test set, which was not used for model training, but only for model evaluation.

## 5. Experimental Results and Analysis

### 5.1. Performance Evaluation Metric

To evaluate the reliability of the proposed deep-learning-based prediction model, the difference between the actual speed and the predicted speed was examined. The mean absolute percentage error (*MAPE*) was calculated using Equation (1):

(1) $$MAPE\left(\%\right)=\frac{100}{T}\sum_{t=1}^{T}\frac{\left|y_{t}-f_{t}\right|}{y_{t}}$$

where:

$$t\;:\mathrm{time}\;\mathrm{index},\;t\in 0,\;\ldots ,\;T\;y_{t}\;:\;\mathrm{actual}\;\mathrm{value}\;f_{t}\;:\;\mathrm{predicted}\;\mathrm{value}.$$

*MAPE* represents the mean absolute error from the actual value and it is suitable for the intuitive evaluation of a situation in which there are significant fluctuations [23]. Considering the nature of a field of traffic where the accurate prediction of low-speed traffic, such as during congestion, is critical, *MAPE* is used as the error metric for the performance evaluation since it is highly sensitive to the prediction of relatively small values.

### 5.2. Performance Analysis of the Suggested Deep Learning Model

**Figure 15:**
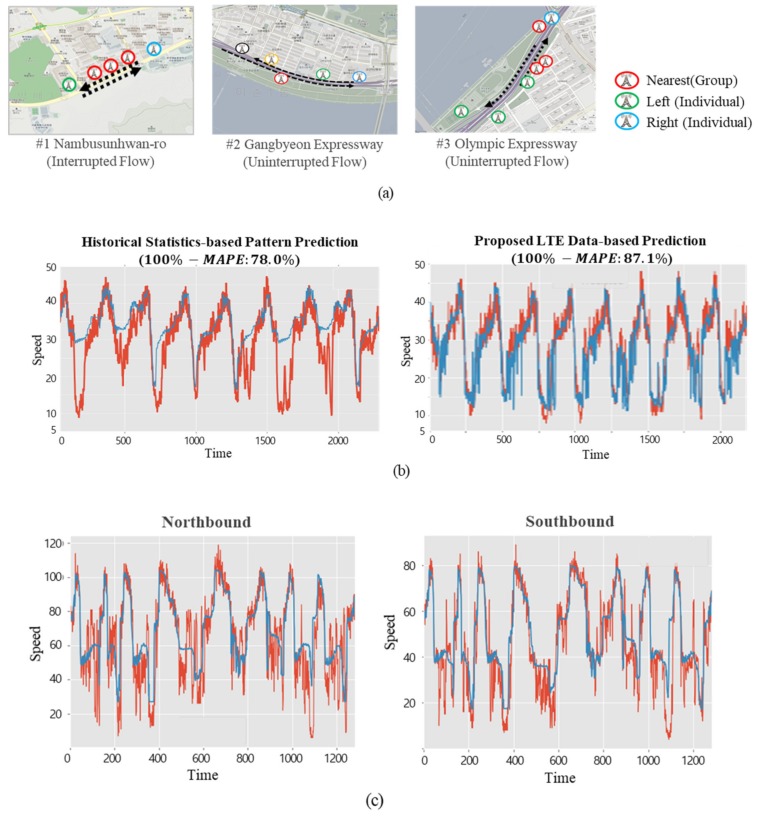

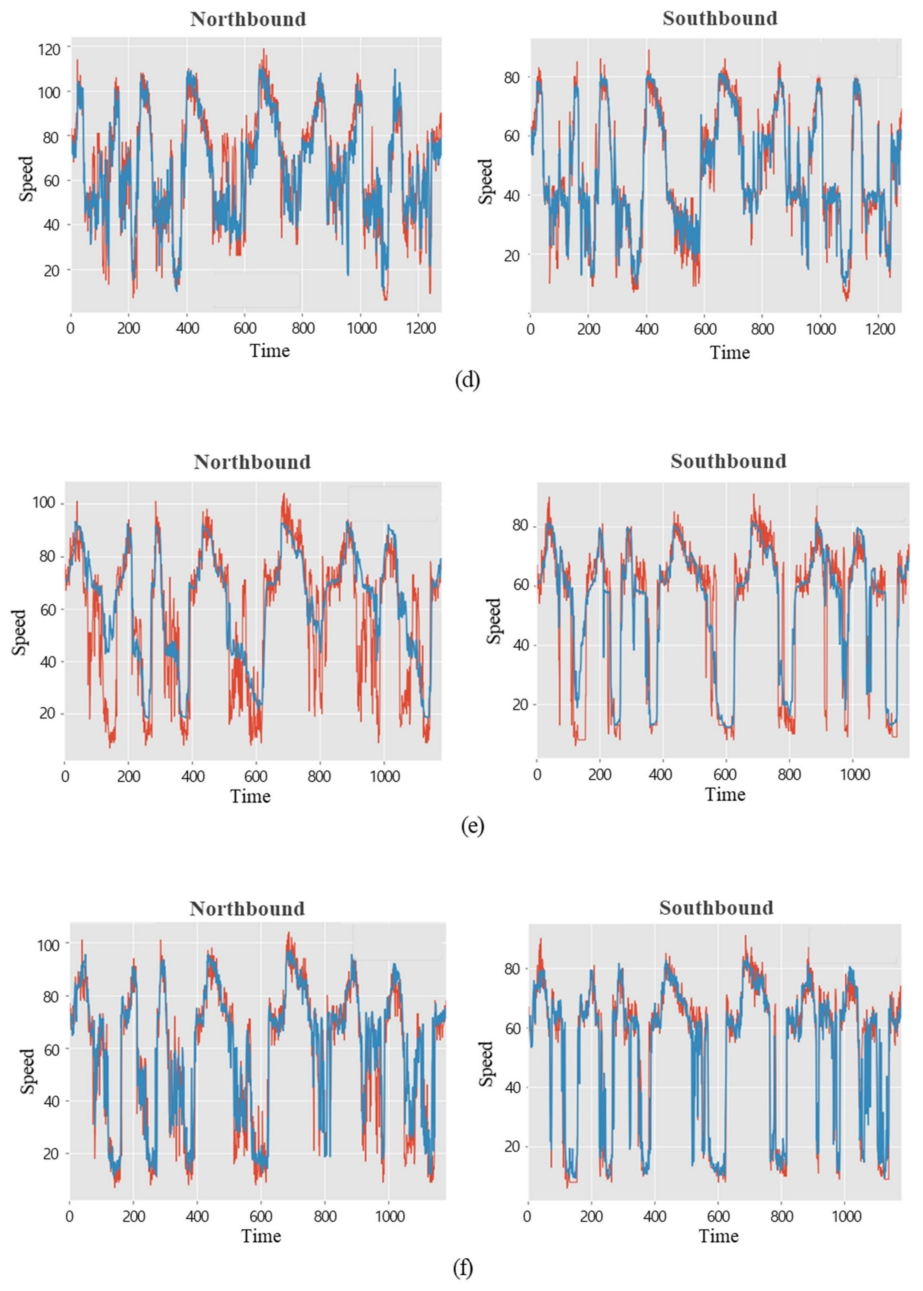
Performance comparison results (red line: actual value, blue line: predicted value.) (**a**) Test regions, (**b**) historical-statistics-based pattern versus proposed LTE data-based prediction in test region #1, (**c**) historical-statistics-based pattern result in test region #2 (100% − *MAPE*: 67.9%), (**d**) proposed LTE-data-based prediction in test region #2 (100% − *MAPE*: 83.4%), (**e**) historical-statistics-based pattern result in test region #3 (100% − *MAPE*: 65.5%), and (**f**) proposed LTE-data-based prediction in test region #3 (100% − *MAPE*: 82.3%).

To evaluate the performance of the proposed deep-learning-based prediction model, as shown in Figure 15, a comparison between the existing statistics-based pattern method and the deep-learning-based prediction using the LTE access data was made for one interrupted road and one uninterrupted road in Seoul. LTE access data from one month was used to create the deep learning model; the traffic speeds of the selected roads were predicted for every five minutes for 10 days, and the differences from the actual speeds were compared using *MAPE*. As shown in Figure 15, 100% − *MAPE* of the suggested deep learning was 84.3%, which was much higher than the existing statistics-based pattern prediction of 70.5%.

### 5.3. Analysis of Road Traffic Speed Predictions in Areas with Different Traffic Flows

**Figure 16:**
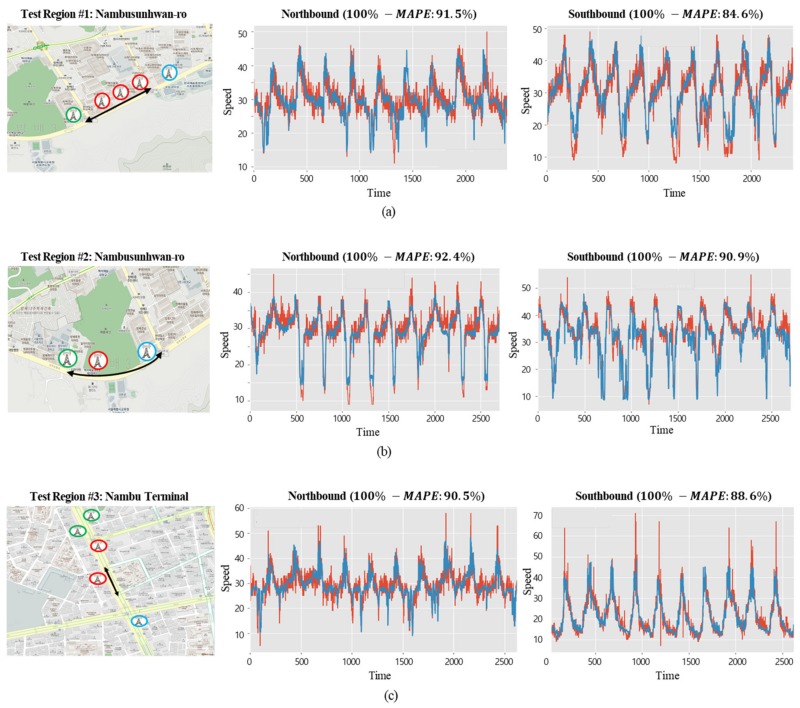
Prediction results of interrupted flow (red line: actual value, blue line: predicted value).

**Figure 17:**
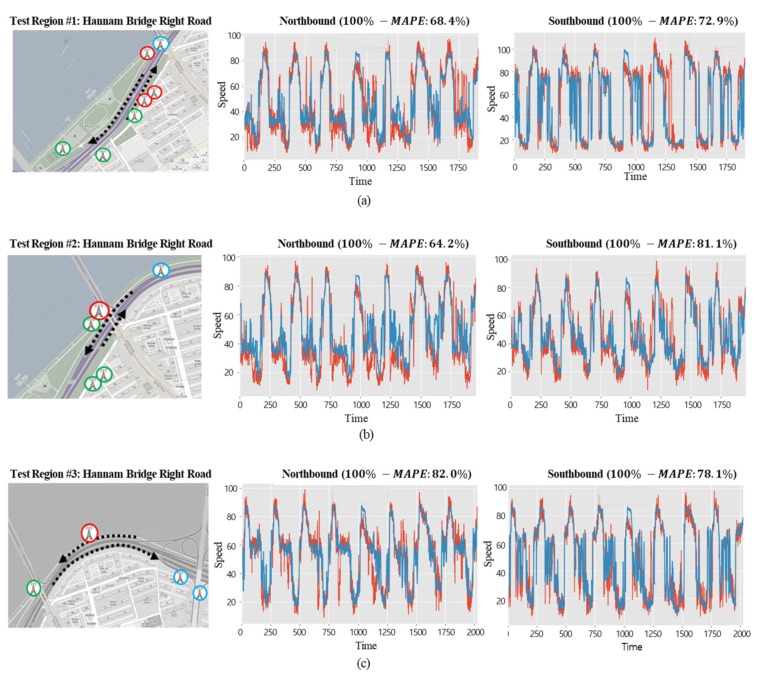
Prediction results of uninterrupted flow (Olympic Expressway) (red line: actual value, blue line: predicted value).

The performance of the suggested deep learning model was evaluated by making predictions for an interrupted road and an uninterrupted road, each having different traffic flows. The characteristics of an interrupted road, as in a downtown area, are as follows: there are several buildings adjacent to the road, pedestrians have a significant influence, and traffic lights are present at each intersection. On the other hand, for the uninterrupted road, such as an urban expressway, there are few buildings adjacent to the road and speed limits are higher than for downtown roads. To evaluate the performance of the suggested deep learning model in areas having different properties, as described in Section 5.2, LTE access data from one month was used to create the deep learning model. Then, the traffic speeds of the selected roads were predicted every five minutes for 10 days, and their differences from the actual speeds were compared with accuracy. Figure 16 shows the predicted speeds of the northbound and southbound lanes of the roads in three downtown areas: the average (100% − *MAPE*) was 89.8%. Figure 17 shows the predicted speeds of the northbound and southbound lanes of the uninterrupted road (the Olympic Expressway) in three areas, where the average (100% − *MAPE*) was 74.5%, relatively lower than downtown and different from what was expected. From the analysis of such variances, it was shown that in the downtown area, the number of directions and roads covered by a single base station was small since the cells were divided in the form of a lattice, whereas the uninterrupted road was rather complicated and consisted of many types of road, such as crossing roads and junctions, so it was harder to make a prediction.

## 6. Conclusions

This paper proposed a deep-learning-based real-time road traffic prediction method using LTE access data between drivers and base stations. In the past, models based on historical speed statistics were mainly used, but they have issues when there is a change in the road environment, as well as providing low-reliability predictions. Therefore, this paper suggested a method of using a time-series-based road traffic speed learning model based on LTE access data. As a result of the experiment, the prediction accuracy of the proposed method was 84.3%, which was better than the 70.5% from the existing historical-statistics-based pattern. It can be used for roads where the environment has changed; moreover, even on roads where the collection of traffic data is invalid (such as in a radio shadow area), it is possible to directly enter real-time wireless communications data into the traffic speed learning model to predict the traffic speeds on the road in real time, in turn raising the accuracy of the real-time road traffic predictions. In addition, in the case of the existing handover method, there is an issue with speed measurement that can be biased by some users, such as stopped drivers or outliers. However, such issues can be resolved since the suggested method generates a road traffic speed learning method that is generalized based on wireless communications access data without handovers. With the proposed deep-learning-based real-time road traffic prediction system, it was possible to generate information about the road and traffic by using the predicted real-time speed, and even to predict unexpected situations, which at the present, can only be learned via reports from a citizen or the police.

In this paper, a model was constructed for each region with similar LTE connection characteristics, but it is necessary to extend it to further study with a generalized prediction model that considers the base station radiation angle or the number of lanes. In addition, although a 4G LTE network was used as the wireless communications connection technology, 5G is currently being distributed. Due to the characteristics of 5G network, it will be installed as base stations at narrower intervals than LTE network base stations, such that the number of connections around roads can be collected more accurately. Therefore, it is necessary to verify the effect by applying the proposed method to the 5G connection data.

## Figures and Tables

**Table 1:** S1 application protocol (S1AP) data schema (gray box: S1AP characterization factor). (MME: Mobility Management Entity, TMSI: Temporary Mobile Subscriber Identity, RLC: Radio Link Control, SRMO: Service Request Mobile Originated, SRMT: Service Request Mobile Terminated, TAU: Tracking Area Update, S1HO: S1 Handover, ESRMO: Extended-SRMO, ESRMT: Extended-SRMT, QCI: QoS Class Index, NAS: Non Access Stratum, PDU: Protocol Data Unit, eNB: Evolved Node B, SGW: Serving Gateway, TAC: Tracking Area Code, PCS: Physical Coding Sublayer).

Column	Description	Remarks
imsi	IMSI value	User unique identification code
mme_cd	MME code value	
tmsi	TMSI value	MME code + TMSI
start_time	S1 call process start time	
end_time	RLC or reset time	
setup_time	Attach request to attach complete processing time	
cal_time	end_time–start_time	
sbt_bts_cd	Alternate base station code	
tp	1: Attach, 2: SRMO, 3: SRMT, 4: TAU, 5: S1HO, 6: Detach, 7: ESRMO, 8: ESRMT	RRC connection detail function type
rrc_estab_cause	RRC establish cause value	RRC connection type
qci	QCI value	Service priority index
reslt	0: success, 1: fail, 2: reset, 3: warning, 4: ODB	
rsn	Defined reject message in NAS PDU	
mbl_ip_adr_inet	Mobile IP address	IPv4 or IPv6
enb_ip	eNB IP address	
mme_ip	MME IP address	
sgw_ip	SGW IP address	
tac	TAC	
cell_id	Cell ID	
pcs_ip	PCS IP	
pagng_stat	1: primary paging,	1: SP(SMRT_TIME-Paging_TIME) < 3 sec
	2: secondary paging,	2: 3 sec ≤ SP < 6 sec
	3: tertiary paging,	3: 6 sec ≤ SP < 10 sec
	4: etc.	4: 10 sec ≤ SP

**Table 2:** S1AP RRC connection type.

RRC Connection Type (S1AP-“rrc_estab_cause”)	Connection Detail Function Type (S1AP-“tp”)
Mobile originating signaling (when the terminal wakes up for signal processing)	Attach: Initial access to MME TAU: Update location when TA changes Detach
Mobile originating data (when the terminal wakes up for data transmission)	SMRO: When a data service is requested E-SMRO
Mobile termination access (when the terminal wakes up due to an incoming packet)	SMRT E-SMRT

**Table 3:** QoS class index (QCI) value for bearers. (TCP: Transmission Control Protocol, WWW: World Wide Web, FTP: File transfer protocol)

QCI	Resource Type	Priority	Packet Delay Budget (ms)	Packet Error Loss Rate	Example Services
1	GBR	2	100	10^−2^	Conversational voice
2	GBR	4	150	10^−3^	Conversational video (live streaming)
3	GBR	3	50	10^−3^	Real-time gaming
4	GBR	5	300	10^−6^	Non-conversational video (buffered streaming)
5	Non-GBR	1	100	10^−6^	IMS signaling
6	Non-GBR	6	300	10^−6^	Video (buffered streaming)
7	Non-GBR	7	100	10^−3^	Voice, video (live streaming), interactive gaming
8	Non-GBR	8	300	10^−6^	TCP-based (WWW, email, FTP); privileged subscriber
9	Non-GBR	9	300	10^−6^	TCP-based (WWW, email, FTP); non-privileged subscriber
